# Non-lysosomal Activation in Macrophages of Atlantic Salmon (*Salmo salar*) After Infection With *Piscirickettsia salmonis*

**DOI:** 10.3389/fimmu.2019.00434

**Published:** 2019-03-19

**Authors:** Diego Pérez-Stuardo, Jonathan Morales-Reyes, Sebastián Tapia, Diego E. Ahumada, Allison Espinoza, Valentina Soto-Herrera, Bernardo Brianson, Valentina Ibaceta, Ana M. Sandino, Eugenio Spencer, Eva Vallejos-Vidal, Felipe E. Reyes-López, Jorge Valdés, Sebastián Reyes-Cerpa

**Affiliations:** ^1^Centro de Genómica y Bioinformática, Facultad de Ciencias, Universidad Mayor, Santiago, Chile; ^2^Consorcio de Sanidad Acuícola, Ictio Biotechnologies S.A., Santiago, Chile; ^3^Centro de Biotecnología Acuícola, Universidad de Santiago de Chile, Santiago, Chile; ^4^Department of Cell Biology, Physiology and Immunology, Universitat Autonoma de Barcelona, Barcelona, Spain

**Keywords:** Atlantic salmon (*Salmo salar*), Piscirickettsiosis, immune evasion mechanisms, macrophages, lysosome, proteolytic activity

## Abstract

*Piscirickettsia salmonis* is a facultative intracellular pathogen and etiological agent of the systemic disease salmonid rickettsial septicemia. It has been suggested that *P. salmonis* is able to survive in host macrophages, localized within a vacuole like-compartment which prevents lysosomal degradation. However, the relevant aspects of the pathogenesis of *P. salmonis* as the host modulation that allow its intracellular survival have been poorly characterized. In this study, we evaluated the role of lysosomes in the response to *P. salmonis* infection in macrophage-enriched cell cultures established from Atlantic salmon head kidneys. Bacterial infection was confirmed using confocal microscopy. A gentamicin protection assay was performed to recover intracellular bacteria and the 16S rDNA copy number was quantified through quantitative polymerase chain reaction in order to determine the replication of *P. salmonis* within macrophages. Lysosomal activity in Atlantic salmon macrophage-enriched cell cultures infected with *P. salmonis* was evaluated by analyzing the lysosomal pH and proteolytic ability through confocal microscopy. The results showed that *P. salmonis* can survive ≥120 h in Atlantic salmon macrophage-enriched cell cultures, accompanied by an increase in the detection of the 16S rDNA copy number/cell. The latter finding suggests that *P. salmonis* also replicates in Atlantic salmon macrophage-enriched cell cultures. Moreover, this bacterial survival and replication appears to be favored by a perturbation of the lysosomal degradation system. We observed a modulation in the total number of lysosomes and lysosomal acidification following infection with *P. salmonis*. Collectively, the results of this study showed that infection of Atlantic salmon macrophages with *P. salmonis* induced limited lysosomal response which may be associated with host immune evasion mechanisms of *P. salmonis* that have not been previously reported.

## Introduction

*Piscirickettsia salmonis* (*P. salmonis*) is the etiological agent of the systemic disease termed Piscirickettsiosis or salmonid rickettsial septicemia, which mostly affects salmonid species in saltwater (1), although it has also been identified in freshwater salmonid cultures (2). *P. salmonis* was first reported in Chile in 1989 as a pathogenic agent in coho salmon (*Oncorhynchus kisutch*) and characterized as a Gram-negative bacterium, non-motile, unencapsulated, pleomorphic, usually coccoid, with a diameter between 0.2 and 1.5 μm (1, 3, 4). *P. salmonis* is an intracellular pathogen, classified phylogenetically as a *Gammaproteobacteria* in the family *Piscirickettsiaceae*, and is closely related to *Legionella, Francisella*, and *Coxiella* (1).

Notably, infection with *P. salmonis* is responsible for high mortality rates only in Chile. In other countries (e.g., Norway, Canada, and Ireland), outbreaks have been reported with limited impact (5, 6). In Chile, the management of Piscirickettsiosis is a national priority given its highly aggressive nature, recurring outbreaks, and wide dispersion among other cultivated salmonid species (7–9). In fact, the National Fisheries Service (SERNAPESCA, Servicio Nacional de Pesca) has identified *P. salmonis* as the most serious health concern facing the Chilean salmon industry (10). The SERNAPESCA has established a monitoring and control program to reduce the impact of salmonid rickettsial septicemia (9). Despite the great impact that *P. salmonis* has had on the aquaculture industry, key aspects of its biology, pathogenesis, and virulence remain poorly understood, significantly hampering the strategies for its control (11, 12). It has been reported that once *P. salmonis* has colonized, the host, it survives and replicates in vacuoles of macrophage-like cells that do not merge with lysosomes (11, 13, 14). Moreover, infection of macrophages with *P. salmonis* induces an anti-inflammatory milieu, probably involved in the development of its bacterial virulence mechanism to ensure replication and survival (15).

Macrophages are part of the first-line cell defense against bacterial infection, specializing in phagocytosis, destruction of foreign microorganism, and presentation of antigens (16, 17). The impact of an infection depends on the balance between the capacity of macrophages to recognize and destroy bacterial pathogens and the ability of pathogens to disrupt the functions of these macrophages (18). Infection of macrophages using different molecular effectors to undermine macrophage signaling is a common strategy adopted by intracellular pathogens to evade the immune system and ensure systemic spread into their hosts. In this way, different bacterial pathogens use diverse strategies to subvert the functions of macrophages. One of the most important strategies is the evasion of phagolysosomal degradation (11, 18). Maturation of the phagosome that fuses with lysosomal compartments—containing an acid and hydrolytic lumen with enzymes responsible for the eradication of internalized bacteria—is critically linked to the destruction of pathogenic bacteria (19, 20). Therefore, interference with the phagosomal maturation is essential for bacteria to enable their presence and replication within macrophages. Importantly, this pathogen strategy has already been reported in bacteria closely related to *P. salmonis*, including *Legionella pneumophila* (*L. pneumophila*), and *Coxiella burnetii* (*C. burnetii*) (21, 22).

Gomez et al. (11), described that *P. salmonis*—along with the phylogenetically-related bacteria *L. pneumophila* or *C. burnetii*—possess the deficient in organelle trafficking/intracellular multiplication (Dot/Icm) secretion system genes classified as type IVB secretion system (TIVBSS) or an equivalent to achieve a productive infection. The Dot/Icm proteins form part of a specialized secretion system encoded by the *dot/icm* genes and constitute the major virulence mechanism of *L. pneumophila* and *C. burnetii* responsible for their intracellular survival and multiplication (23–25). During infection, *L. pneumophila* evades phagosome-lysosome fusion and phagosome acidification. Although subtly increased, this process is delayed for ≥16 h (26). Moreover, *L. pneumophila* has the ability to alter the maturation of the endocytic vacuole in which it initially resides, allowing the establishment of an organelle within phagocytic host cells that supports replication (27). Conversely, *C. burnetii* resides in an acidified lysosome-like compartment, in which it can replicate in the presence of several lysosomal proteins such as v-ATPase (22). To the best of our knowledge, there is limited evidence regarding the lysosomal acidification and functionality of Atlantic salmon macrophages infected with *P. salmonis*. The availability of such evidence may assist in unraveling the host-pathogen interaction mechanisms associated with *P. salmonis* infection.

The aim of this study was to determine whether the lysosomal activity in Atlantic salmon macrophages is modulated by infection with *P. salmonis*.

## Materials and Methods

### Experimental Fish

Atlantic salmon (*Salmo salar*) with an average weight of 55 ± 15 g were obtained from a local farm and maintained in tanks with freshwater at a biomass of 10–12 Kg m^−3^ and controlled temperature (14–16°C) with continuous aeration. Water quality parameters, such as pH, oxygen, and the levels of nitrogen compounds (i.e., nitrate, nitrite, and ammonia) were monitored daily and maintained at constant values. The fish were fed with commercial pellets twice daily (Golden Optima, Biomar, Puerto Montt, Chile), and acclimatized for ≥3 weeks prior to the experiments. The maintenance of fish was performed in accordance with the ethical standards of the Institutional Ethics Committee of Universidad de Santiago de Chile (approved in internal report n°364) and the relevant legislation in force.

### Isolation of Macrophages and Cell Culture

Macrophage-enriched cell cultures were obtained from Atlantic salmon head kidneys as described by Braun-Nesje et al. (28) with slight modifications. Briefly, the head kidney of fish was aseptically extracted and disaggregated using a cell strainer (pore size: 70 μm) (BD Falcon, Seaton Delaval, England) on a 60 × 15 mm culture plate (BD Falcon) in the presence of Leibovitz medium (L-15; Hyclone, Thermo Scientific, Massachusetts, USA) supplemented with 2-mercaptoethanol 36 μM (2-ME; Gibco, Thermo Scientific), fetal bovine serum 3% (v/v) (FBS; Hyclone, GE Healthcare Life Sciences, Utah, USA), penicillin 200 U/mL, streptomycin 200 μg/mL (Corning, New York, NY, USA), amphotericin B 2.5 mg/mL (Corning), and sodic heparin 24.7 UI/mL (Leo Pharma, Ballerup, Denmark) (supplement 1). mechanical disaggregation was performed until a homogeneous cell suspension was obtained. The leukocyte fraction was isolated through a discontinuous gradient in densities of 34% and 51% of Percoll (GE Healthcare) diluted in miliQ water and Hank's Balanced Salt Solution (HBSS 10X, Gibco). The cell suspension was deposited on the discontinuous Percoll gradient and the column was centrifuged for 40 min at 400 *g* at 16°C. The leukocyte interface was recovered and resuspended in L-15 medium (supplement 1). To eliminate the traces of Percoll, the cell suspension was centrifuged twice for 7 min at 250 *g* at 16°C. The cell suspension was placed on 12-well plates at 40,000 cells/cm^2^ in L-15 medium supplemented with 2-ME 36 μM, FBS 10% (v/v) (Hyclone), penicillin 200 U/mL, streptomycin 200 μg/mL (Corning), and amphotericin B 2.5 mg/mL (Corning) (supplement 2) at 16°C. At day 1, the primary culture was washed with phosphate-buffered saline (PBS) 1X, non-adherent cells were discarded to enrich the monocytic/macrophage adherent population, and cultivated in L-15 medium containing supplement 2. At days 3, 5, and 7, the cultured cells were washed with PBS 1X, the non-adherent cells were discarded, and the medium of the cell cultures was replaced by L-15 medium supplemented with 2-ME 36 μM and FBS 10% (v/v) (Hyclone) (supplement 3).

### Cultivation and Propagation of *P. salmonis*

Culture and propagation of *P. salmonis* (strain 9734, ETECMA, Puerto Montt, Chile) was performed in salmonid cell line CHSE-214 (ATCC N°CRL-1682) as previously described by Fryer et al. (29). The CHSE-214 cell line was maintained in minimal essential medium (MEM; Corning) FBS 10% (v/v) (Hyclone), HEPES buffer 10 mM (Corning), and non-essential amino acids 1% (v/v) (Corning) (supplement 4) at 16°C. The infection was observed through conventional inverted microscopy (Motic AE31E) 4–6 days post-infection (dpi) to determine the cytopathic effect on cells (30). The bacteria were quantified using a Petroff–Hausser chamber (Hausser Scientific, Pennsylvania, USA) according to the instructions provided by the manufacturer.

### Inactivation of *P. salmonis*

*P. salmonis* was extracted from the infection supernatant of CHSE-214 cells with evident cytopathic effect. Cellular debris was eliminated through centrifugation for 5 min at 500 *g*. Subsequently, *P. salmonis* was centrifuged for 15 min at 7,500 *g* at 16°C. The pellet was resuspended in 4% paraformaldehyde (PFA; Sigma Aldrich, Missouri, USA) diluted in PBS 1X and incubated overnight at 4°C. Finally, the bacterial suspension was centrifuged for 15 min at 7,500 *g* at 16°C, the supernatant was discarded, and the pellet was resuspended in L-15 medium containing supplement 3.

### Detection of **P. salmonis** Using Quantitative Polymerase Chain Reaction (qPCR)

The gene encoding for 16S rRNA (Fw: 5′-AGG-GAG-ACT-GCC-GGT-GAT-A-3′; Rv: 5′-ACT-ACG-AGG-CGC-TTT-CTC-A-3′) was amplified as described by Karatas et al. to detect the presence of *P. salmonis* in the infected cell cultures (31). Genomic DNA was obtained using the Wizard™ Genomic DNA Purification kit according to the instructions provided by the manufacturer. The PCR amplification was performed using the PowerUp™ SYBR® Green master Mix (Thermo Scientific) according to the instructions provided by the manufacturer. The primers were added to a final concentration of 0.4 μM, and 12 ng of template were used. The qPCR was performed on a QuantStudio 3 Real-Time PCR system (Thermo Scientific). The quantification of 16S rDNA copies was determined through interpolation from the standard curve with the cycle threshold (Ct) value obtained for each sample. The results are expressed as 16S rDNA copy/cell.

#### Fluorescein Isothiocyanate (FITC) Staining for **P. salmonis**

**Figure 1:**
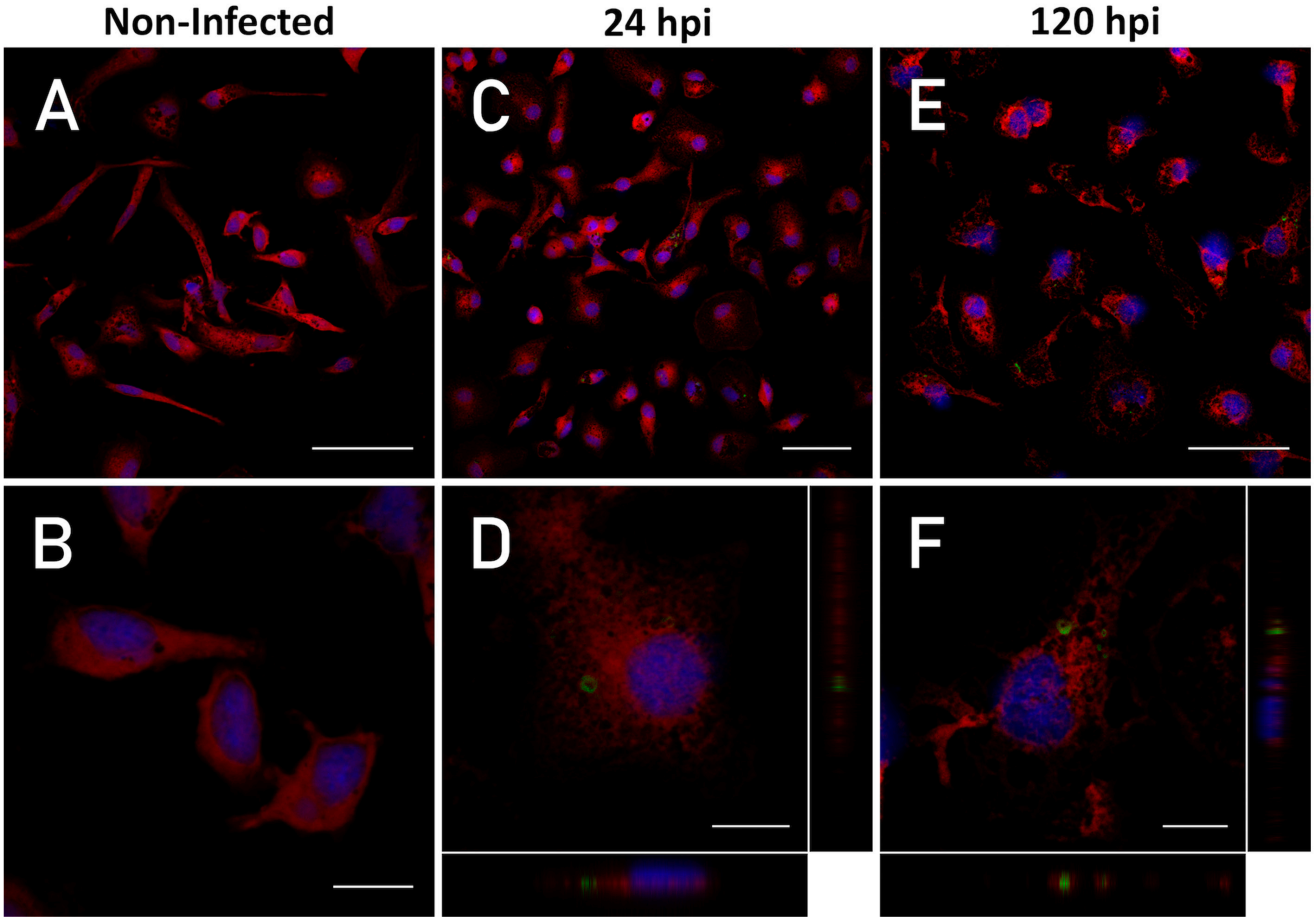
Intracellular detection of *P. salmonis*. Macrophage-enriched cultures obtained from Atlantic salmon head kidneys were incubated with *P. salmonis*-FITC at a MOI of 0.5 bacteria/cell for 24 and 120 hpi in L-15 supplemented medium. The cellular nucleus (stained with DAPI; blue), cell cytoplasm (stained with CMPTX; red), and *P. salmonis*-FITC (green) are represented. The intracellular location of *P. salmonis*-FITC was determined by confocal microscope orthogonal image analysis from the z-stack obtained. **(A,B)** Non-infected macrophages (scale bar 50 and 10 μm, respectively). **(C,D)** Macrophages at 24 hpi (scale bar 50 and 10 μm, respectively). **(E,F)** Macrophages at 120 hpi (scale bar 50 and 10 μm, respectively).

In order to monitor the *P. salmonis* infection into macrophage-enriched cell cultures, we stained the bacteria using FITC (Thermo Scientific). *P. salmonis* was extracted from the infection supernatant of CHSE-214 cells with evident cytopathic effect and centrifuged for 15 min at 7,500 *g* at 16°C. Subsequently, the bacteria were incubated for 30 min with FITC 100 μg/mL diluted in L-15 medium without supplement. Finally, the bacteria were centrifuged for 15 min at 7,500 *g* at 16°C and resuspended in L-15 medium without supplement. The viability of *P. salmonis* was determined by comparison of bacterial growth in enriched blood agar plates (32) with non-stained *P. salmonis* (Figure 1). Growth of bacterial colonies was analyzed for the specific detection of 16S rDNA using real time PCR (31) and morphology as previously described by Mauel et al. (32).

### *P. salmonis* Infection

Macrophage-enriched cell cultures from Atlantic salmon head kidneys were incubated for 30 min with 10 μM Cell Tracker Red (CMPTX; Thermo Scientific) diluted in L-15 without supplements. Subsequently, the cells were washed thrice with PBS 1X and incubated with *P. salmonis*-FITC stained at a multiplicity of infection (MOI) of 0.5 bacteria/cell (previously quantified using a Petroff–Hausser chamber), and cultured in L-15 medium containing supplement 3. Cells were incubated for 24 and 120 h post-infection (hpi). Subsequently, the cell culture was fixed with 4% PFA for 10 min and washed thrice with PBS 1X. The nucleus was stained with 4′,6-diamidino-2-phenylindole, dihydrochloride (DAPI) 0.1 μg/mL for 1 min and the cells were washed twice with PBS 1X and once with MiliQ water to wash the residuary salts. Subsequently, the cells were mounted on slides using the mounting solution Fluoromount™ (Thermo Scientific). Micrographs were obtained using a confocal microscope Zeiss LSM 710, the images were processed and analyzed using the Fiji software (33).

### Gentamicin Protection Assay

In order to recover the intracellular bacterium from the infected macrophage-enriched cell cultures, a gentamicin protection assay was performed. Briefly, macrophage-enriched cell cultures were infected with *P. salmonis* at a MOI of 10 bacteria/cell for 24 and 120 h. Recovery of the intracellular bacterium was performed following the protocol described by Rodriguez et al. (34) Imarai et al. (35), and Fast et al. (36), with modifications. Macrophage-enriched cell cultures infected with *P. salmonis* were incubated with gentamicin (100 μg/mL) for 60 min to eliminate bacteria from the extracellular environment. After 3–5 washes with cold PBS 1X, saponin (Sigma) was added (1% p/v in PBS 1X) for 15 min at 16°C to permeabilize the cells. Finally, the homogenized cells were diluted in PBS 1X and centrifuged at 6,000 g for 10 min at 4°C. The supernatant was discarded and the pellet was resuspended in 100 μL of PBS 1X. Genomic DNA from the homogenized cells was extracted using the Wizard™ Genomic DNA Purification kit according to the instructions provided by the manufacturer. Quantification of intracellular *P. salmonis* recovered from head kidney cell cultures was performed through specific qPCR absolute quantification using the set of primers described by Karatas et al. (31). The supernatant used for the gentamicin treatment as well as the PBS used for each wash were recovered and subsequently centrifuged at 6,000 g for 10 min at 4°C to eliminate any viable extracellular bacteria after treatment with gentamicin or the subsequent washes. This process was performed to prevent an overestimation of the intracellular bacteria. The obtained pellets were resuspended in 100 μL PBS. Part of these suspensions (50 μL) was plated in CHAB agar and cultivated by 14 days at 20°C. The remaining 50 μL were used to extract genomic DNA for the qPCR amplification of 16S rDNA with the same specific primers mentioned above.

### Evaluation of Lysosomal Acidification

Evaluation of lysosomal acidification in *P. salmonis*-infected macrophage-enriched cell cultures was performed by fluorescence analysis using the LysoSensor™ Yellow/Blue probe (LSYB; Thermo Scientific). This ratiometric probe can be used to measure the pH of acidic organelles. The LysoSensor™ dye produces blue and yellow fluorescence in neutral and acidic environments, respectively. Therefore, a fluorescence shift from blue (maximum emission at 430 nm) to yellow (maximum emission at 535 nm) indicates an increase in lysosomal pH (37).

The macrophage-enriched cell cultures were established in 12-well plates with glass coverslips and infected with *P. salmonis* at a MOI of 10 bacteria/cell for 1 h in L-15 medium containing supplement 3. After incubation, the cell cultures were washed once with PBS 1X and incubated with L-15 containing supplement 3. In order to determine the short-term effect on lysosomal acidification in *P. salmonis*-infected macrophage-enriched cell cultures, a lysosomal acidification analysis was performed at 3, 6, and 24 hpi. A non-infected and an incubation with inactivated *P. salmonis* conditions were simultaneously performed as internal experimental controls. At each time point, the infected cells were incubated with LSYB 10 μM for 5 min. Subsequently, the cells were washed thrice with PBS 1X and fixed with 4% PFA (p/v) (Sigma Aldrich) for 10 min, followed by three additional washes with PBS 1x. Finally, the nucleus was stained with propidium iodide (PI) 1 μg/mL (incubation for 1 min) and the cells were washed twice with PBS 1X and once with MiliQ water to remove the residuary salts. The samples were mounted on slides with Fluoromount™ (Sigma Aldrich) and the images were obtained using a Leica SP8 confocal microscope (Leica, Wetzlar, Germany). Results were obtained by the division of the emission spectrum of the LSYB probe, specifically to obtain an acidic indicator channel (acquired between 500 and 580 nm) and a neutral-basic indicator channel (acquired between 450 and 495 nm). According to instructions provided by the manufacturer, the fluorescence intensity obtained in the acidic indicator channel had to be ≥2-fold than that obtained in the neutral-basic indicator channel to consider a lysosome as acidic. The analysis of lysosomal acidity was performed using the software LAS X (version 3.3.0). Briefly, the fluorescence histogram intensity for each indicator channel was obtained for each lysosome in the samples, and the fluorescence intensity average per count value was calculated using the software. Subsequently, the ratio between the values obtained from the two indicator channels was used as the acidity index. An acidic index ≥2 denoted an acidic pH. Conversely, an acidity index 0<x<2 denoted neutral-basic pH. The number of lysosomes per sample was normalized to the number of cells per sample. For each condition, four random micrographs with a z-stack containing on average 30 cells were obtained. Every lysosome present in the micrograph was analyzed based on the region of interest selected using the software. The micrographs were processed through the software Fiji (33). The results are represented as the number of lysosomes/cell and percentage of acidic lysosomes per condition.

### Evaluation of Lysosomal Activity

In order to determine the short-term effect on the lysosomal activity in *P. salmonis*-infected macrophage-enriched cell cultures, a fluorescence analysis using the DQ™ Green BSA probe was performed. This probe is composed of albumin derivatized with a self-quenching fluorochrome. The degradation of DQ™ Green BSA in acidic lysosomes results in smaller protein fragments than those of isolated fluorophores. Once the quencher is released, brightly fluorescent products are observed. The cleavage of DQ™ Green BSA results in the release of fragments with maximum excitation and emission at 505 and 515 nm, respectively (38–40).

The macrophage-enriched cell cultures were established in 12-well plates with glass coverslips and infected with *P. salmonis* on a MOI of 10 bacteria/cell for 1 h in L-15 medium containing supplement 3. After the incubation, the cell cultures were washed once with PBS 1X and incubated with L-15 containing supplement 3. Subsequently, an analysis of lysosomal activity was performed at 3, 6, and 24 hpi with *P. salmonis*. A non-infected and an incubation with inactivated *P. salmonis* were simultaneously performed as internal experimental controls. Two h prior to reaching each time point (i.e., 3, 6, and 24 hpi), DQ™ Green BSA 10 μg/mL in L-15 medium containing supplement 3 was added to the infected cells. Subsequently, the cells were washed thrice with PBS 1X and fixed using 4% PFA. The nuclei were stained with PI 1 μg/mL as previously described. The samples were mounted on slides using the mounting solution Fluoromount™ (Thermo Scientific). The micrographs were obtained using a Leica SP 8 confocal microscope (Leica), and processed and analyzed using the Fiji software (33). The analysis was performed by obtaining four micrographs with a z-stack to determine the total amount of hydrolytic events per cell that contains in average 30 cells on the micrographs, for each experimental condition and counting every positive event to the fluorescence of DQ™ Green BSA. The data were normalized to the number of cells on the micrograph and the results are represented as the number of proteolytic events/cell.

### Statistical Analysis

Statistical differences in the quantification of *P. salmonis* recovered from macrophage-enriched cell cultures were determined using the one-tailed Mann–Whitney U test. Statistical differences in proteolytic events between the groups analyzed were determined using two-way analysis of variance (ANOVA) with a Dunnett's multiple comparison test. We used the GraphPad Prism v6.0 for Windows software (GraphPad Software Inc.) to calculate the mean and the standard error of the mean (SEM) and perform the statistical tests. A *p* < 0.05 denoted statistical significance.

## Results

### **P. salmonis** Infects Macrophage-Enriched Cell Cultures Obtained From Atlantic Salmon Head Kidneys

Our first aim was to demonstrate that the bacteria invade the immune system, reside, and replicate in macrophage-enriched cell cultures obtained from Atlantic salmon head kidney infected with *P. salmonis*. To detect and visualize *P. salmonis* in the macrophages, the cells were infected with FITC-labeled *P. salmonis*. The simultaneous detection of *P. salmonis*-FITC red-stained cytoplasm and DAPI-stained nucleus on 1.16-μm-thickness cross-sectional images by confocal microscopy showed that, after 24 and 120 h of incubation, bacteria were found in the cytoplasm of macrophages. An orthogonal view of the cells (midplane z section; height: 1.3 μm) confirmed the intracellular localization of *P. salmonis* (Figure 1). Previously, the viability (growth and morphology) of *P. salmonis*-FITC was compared with that observed in non-stained *P. salmonis*. Both bacteria were seeded in enriched-blood agar and—after 10 days of incubation—small single (0.1 mm) circular, entire, white, and convex colonies of *P. salmonis* were visible. Single colonies were selected and specific real time PCR was performed. This analysis yielded a positive detection of 16S rDNA of *P. salmonis* with a unique amplification product (Supplementary Figure 1).

**Figure 2:**
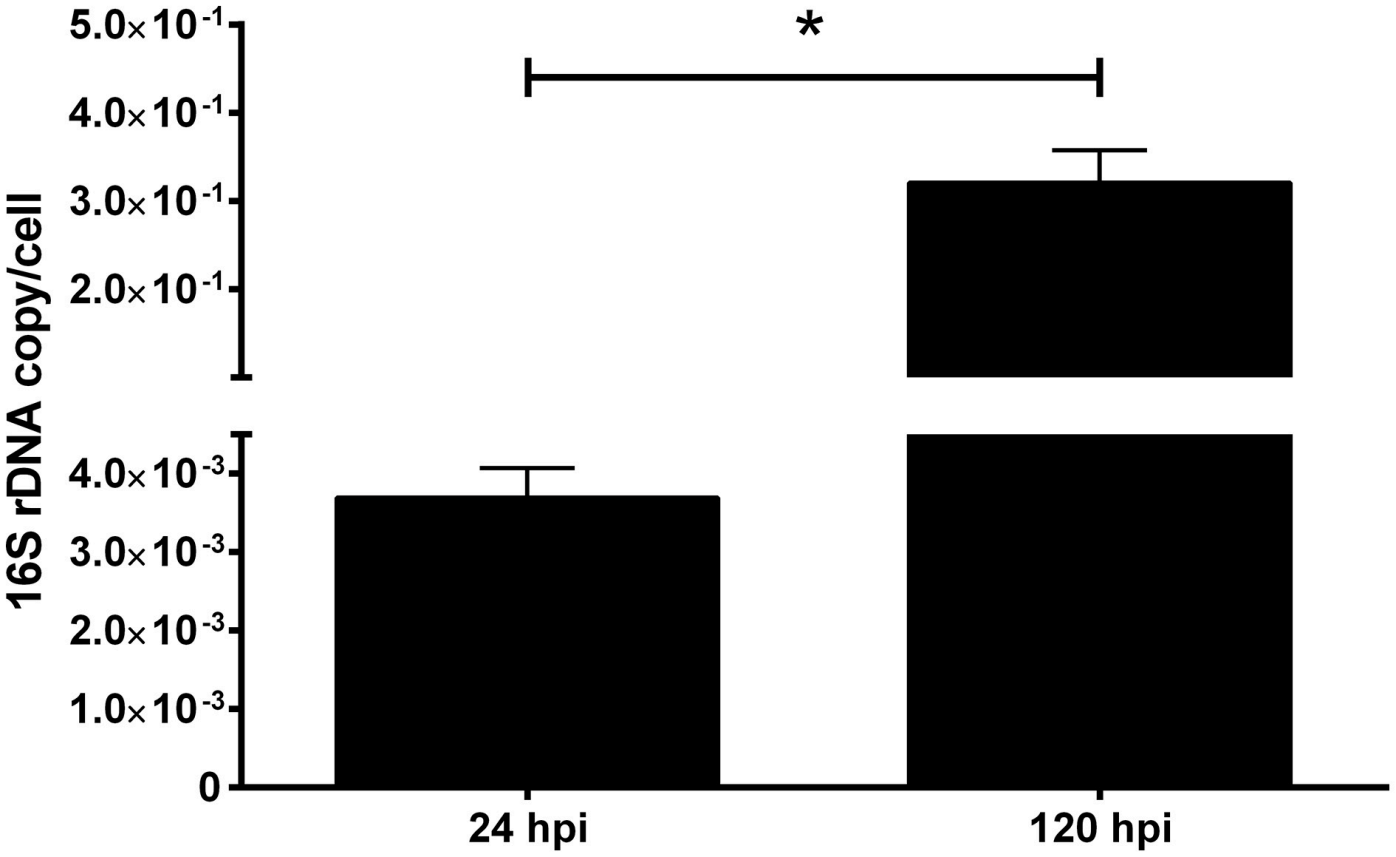
16S rDNA quantification of *P. salmonis* infecting macrophage-enriched cell cultures. Macrophage-enriched cultures obtained from Atlantic salmon head kidneys were incubated with *P. salmonis*-FITC at a MOI of 0.5 bacteria/cell in L-15 supplemented medium. The coding gene of the ribosomal 16S subunit was quantified at 24 and 120 hpi to indirectly evaluate the bacterial proliferation. The data (mean ± SEM) were normalized to the number of cells per sample. The statistical analysis was performed using Student's *t*-test with a Mann–Whitney post-statistical test. Significant difference: *p < 0.05.

In the gentamicin protection assay, the quantification of intracellular bacteria recovered from macrophage-enriched cell cultures reflects a two magnitude orders significant increase in the 16S rDNA copy number/cell between 24 and 120 hpi (Figure 2). The results indicated that *P. salmonis* is able to infect macrophages after 24 hpi, as well as survive and replicate in macrophage-enriched cell cultures after 120 hpi. This evidence suggests that *P. salmonis* may modify the mechanisms associated with pathogen eradication, thus enabling the localization and replication in macrophages.

### Lysosomal Acidification Is Affected in **P. salmonis**-Infected Macrophage-Enriched Cell Cultures

We evaluated the lysosomal acidification of macrophages to determine whether infection with *P. salmonis* favors the modification of the mechanisms associated with pathogen eradication in the first hpi. For this purpose, we incubated macrophage-enriched cell cultures with *P. salmonis* for 1 h.

**Figure 3:**
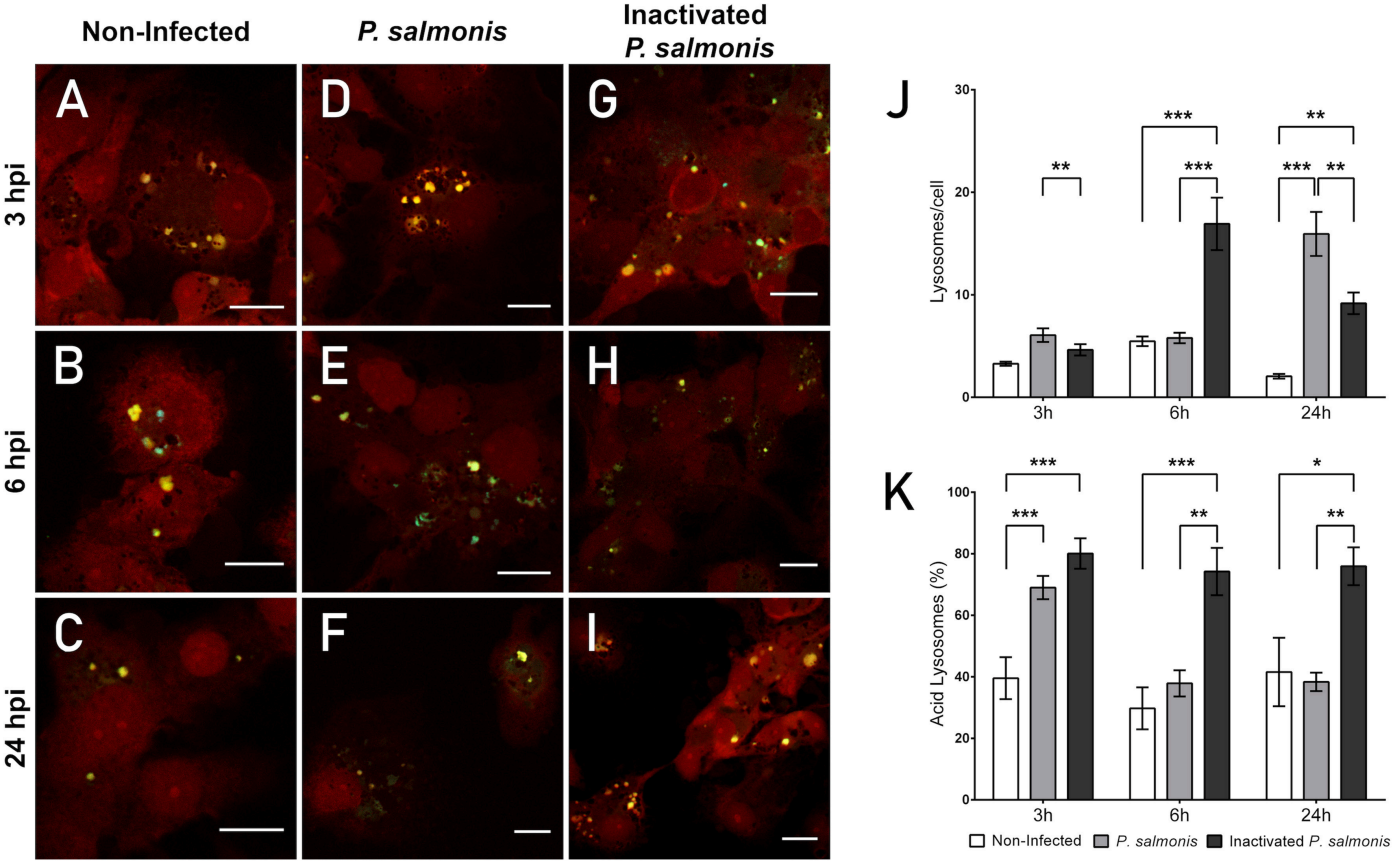
Lysosomal acidification in macrophage-enriched cell cultures infected with *P. salmonis*. Macrophage-enriched cultures obtained from Atlantic salmon head kidneys were incubated with *P. salmonis* at a MOI of 10 bacteria/cell and analyzed at 3, 6, and 24 hpi. The macrophage-enriched cell culture was treated with the LSYB probe (green and blue) to stain lysosomes and analyze their pH. The nucleus was stained with PI (red). **(A–C)** Non-infected macrophages analyzed at 3, 6, and 24 h. Macrophages-enriched cell culture incubated with *P. salmonis* for 3 h **(D)**, 6 h **(E)**, and 24 h **(F)**. Macrophage-enriched cell culture incubated with inactivated *P. salmonis* for 3 h **(G)**, 6 h **(H)**, and 24 h **(I)**. Scale bar: 10 μm **(J,K)**. Lysosomal quantification in macrophage-enriched cell cultures infected with *P. salmonis*. Macrophage-enriched cell culture was infected with *P. salmonis* at a MOI of 10 bacteria/cell and analyzed at 3, 6, and 24 hpi. The lysosomes were stained with the LSYB probe and quantified as acidic lysosomes or neutral-basic (NB) lysosomes. The data were normalized to the number of cells analyzed. Non-infected macrophage-enriched cell culture and incubation with inactivated *P. salmonis* were used as controls for each time point. **(J)** Total number of lysosomes per cell. **(K)** Percentage of acidic lysosomes for each condition. The statistical analysis was performed through parametric ANOVA with a Dunnett's multiple comparison test. Significant differences: *p < 0.05, **p < 0.01, ***p < 0.001.

The confocal microscopy images showed the presence of vesicles with a degree of acidity (Figures 3A–C) in non-infected cells at all time points analyzed—detected by a punctate pattern of yellow/green fluorescence. Blue fluorescence dots were also observed, suggesting the presence of vesicles with a neutral pH. Analysis of the fluorescence emission in *P. salmonis*-infected macrophage-enriched cell cultures showed that, at 3 and 6 hpi, a marginal or no increase was observed in the number of punctate patterns of yellow/green fluorescence compared with that identified in non-infected cells (Figures 3D,E). An increase in the number of punctate patterns of yellow/green fluorescence was observed only at 24 hpi (Figure 3F) vs. that observed for the non-infected macrophage-enriched cell cultures (Figure 3C). These results were corroborated by the quantification of the number of lysosomes/cells in macrophage-enriched cell cultures infected with *P. salmonis*. This analysis showed a slight increase in number compared with that recorded for the non-infected cells at 3 hpi, which only got a significant increase at 24 hpi (15 lysosomes/cell) compared to 3 hpi with *P. salmonis* (6 lysosomes/cell) (Figure 3J). Conversely, following the incubation of macrophage-enriched cell cultures with inactivated *P. salmonis*, the number of punctate patterns of yellow/green fluorescence increased at 3, 6, and 24 hpi (Figures 3G–I) compared with those recorded for non-infected cells (Figures 3A-C) and for those infected with live *P. salmonis* (Figures 3D–F). These results suggest an increase in the number of acidic vesicles against inactivated *P. salmonis*, but not against live *P. salmonis*.

Subsequently, we determined the percentage of acidic lysosomes in macrophage-enriched cell cultures infected with *P. salmonis*. Despite the slight increase observed in the number of lysosomes at 3 hpi, we detected a significant increase in the percentage of acidic lysosomes (Figure 3K). However, the percentage of lysosomes with an acidic pH in macrophage-enriched cell cultures infected with *P. salmonis* decreased with longer infection times (i.e., 6 and 24 hpi) (Figure 3K), despite the higher number of lysosomes/cell observed at 24 hpi (Figure 3J). Conversely, following the incubation of macrophage-enriched cell cultures with inactivated *P. salmonis*, the percentage of acidic lysosomes increased to approximately 80% of all lysosomes evaluated after 3h post-incubation. This increase was significantly different compared with that observed in non-infected cells and slightly superior to that observed in macrophage-enriched cell cultures infected with *P. salmonis* (Figure 3K). The results obtained at 6 and 24 h post-incubation were similar, with approximately 70% acidic lysosomes observed in the samples. This finding represents a significant increase compared with the values obtained for non-infected cells and macrophage-enriched cell cultures infected with *P. salmonis* (Figure 3K). These results indicate that lysosomes are not acidified following the infection of macrophage-enriched cell cultures with *P. salmonis*. Conversely, following the incubation of macrophage-enriched cell cultures with inactivated *P. salmonis*, there is an increase in the percentage of acidic lysosomes along with an increase in the number of lysosomes/cell. This finding suggests that lysosomal activation in macrophage-enriched cell cultures occurs after an appropriate stimulus.

### Infection With **P. salmonis** Diminishes the Hydrolytic Activity in Macrophage-Enriched Cell Cultures

**Figure 4:**
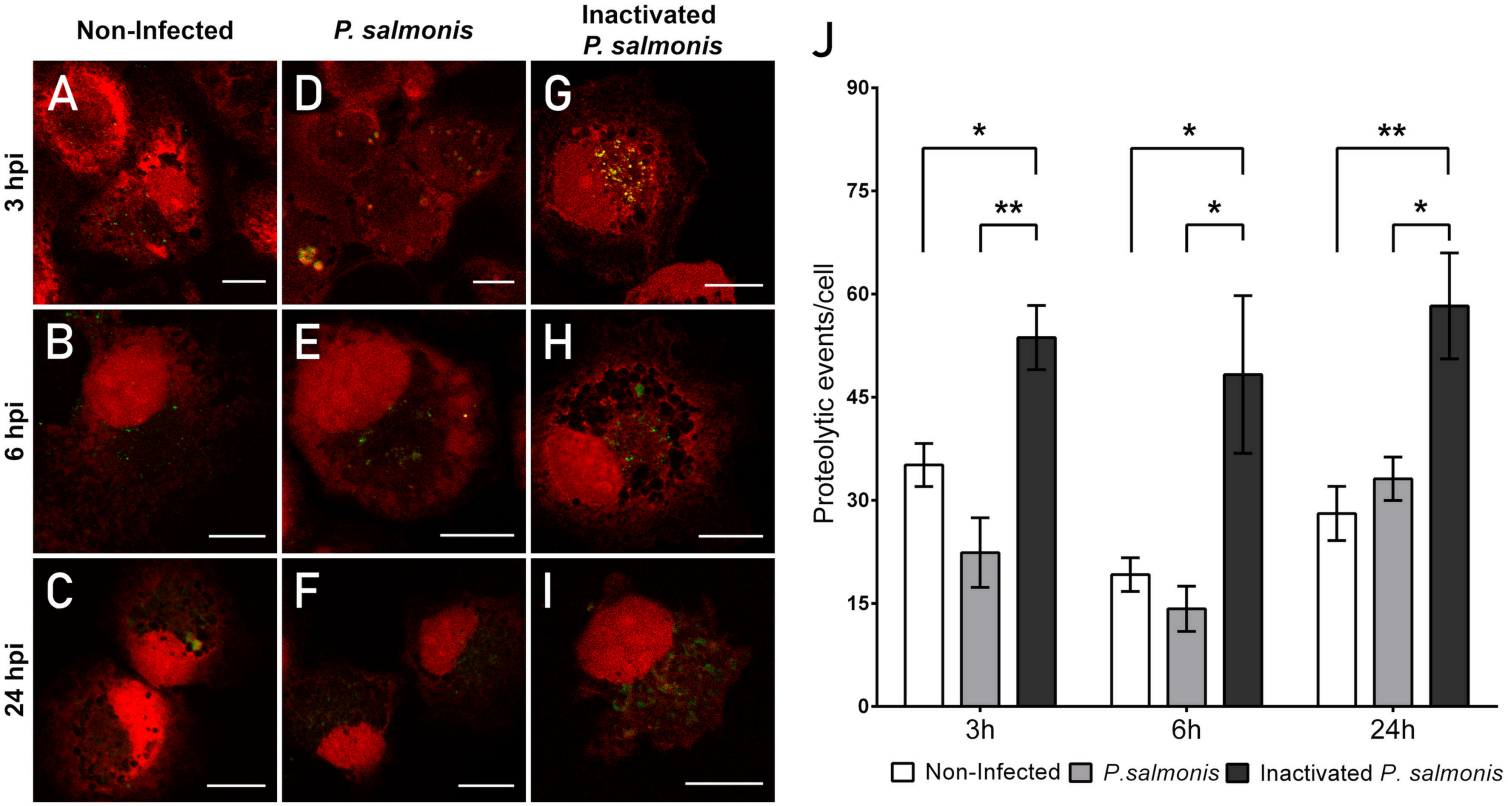
Lysosomal functionality in macrophage-enriched cell cultures infected with *P. salmonis*. Macrophage-enriched cell cultures were infected with *P. salmonis* at a MOI of 10 bacteria/cell and analyzed at 3, 6, and 24 hpi. Cells were treated with the DQ-BSA^TM^ Green probe to stain the proteolytic focusses; the nucleus was stained with PI (red). Non-infected macrophage-enriched cell culture and incubation with inactivated *P. salmonis* were used as controls for each time point. **(A–C)** Non-infected macrophages analyzed at 3, 6, and 24 h. Macrophage-enriched cell culture incubated with *P. salmonis* for 3 h **(D)**, 6 h **(E)**, and 24 h **(F)**. Macrophage-enriched cell culture incubated with inactivated *P. salmonis* for 3 h **(G)**, 6 h **(H)**, and 24 h **(I)**. Scale bar: 10 μm. **(J)** Quantification of proteolytic events in macrophage-enriched cell cultures infected with *P. salmonis*. Macrophage-enriched cell cultures were infected with *P. salmonis* at a MOI of 10 bacteria/cell and analyzed at 3, 6, and 24 hpi. The proteolytic events were detected using the DQ-BSA Green probe and data were quantified and normalized to the number of cells analyzed for each condition. Non-infected macrophage-enriched cell culture and incubation with inactivated *P. salmonis* were used as controls for each time point. The statistical analysis was performed through a parametric ANOVA with a Dunnett's multiple comparison test. Significant differences: *p <0.05, **p < 0.01.

One of the key steps in pathogen eradication is the proteolytic activity of phagocytes. Thus, the proteolytic activity of macrophages was evaluated to determine whether infection with *P. salmonis* modifies the cell host proteolytic activity in the first hpi as a mechanism of immune evasion. The proteolytic activity was assessed using the fluorescent degradative event marker DQ™ Green BSA. According to the results, non-infected macrophage-enriched cell cultures exhibited a low number of fluorescent events at all time points (Figures 4A–C). Interestingly, following the infection of macrophag e-enriched cell cultures with *P. salmonis*, a similar pattern in fluorescence emission from proteolytic events/cell was observed vs. that reported in non-infected cells at all time points (Figures 4D–F). Conversely, following the incubation of macrophage-enriched cell cultures with inactivated *P. salmonis* for 3 h, we observed a greater number of punctate patterns of intense green fluorescence (Figure 4G) vs. that observed in non-infected cells. Similar results were observed at 6 and 24 hpi, with proteolytic events reported by intense fluorescence events (Figures 4H–I). At both time points, greater numbers of events were observed vs. those reported in non-infected cells and macrophage-enriched cell cultures infected with *P. salmonis*.

In order to assess whether *P. salmonis* affects the proteolytic activity of macrophage-enriched cell cultures, we quantified the fluorescent events using confocal microscopy (Figure 4J). At 3 hpi, we observed a non-significant decrease in the number of proteolytic events/cell compared with that reported in non-infected cells (22 vs. 35 proteolytic events/cell, respectively) (Figure 4J). Similar results were observed at 6 and 24 hpi with *P. salmonis*; however, the differences were not significant (Figure 4J). Conversely, following the incubation of macrophage-enriched cell cultures with inactivated *P. salmonis*, we observed a significant increase in the number of proteolytic events/cell compared with that reported in non-infected cells at all time points, reaching 60 proteolytic events/cell at 24 hpi (Figure 4J). Collectively, these results suggest an inactivation of lysosomal activity following the infection of macrophage-enriched cell cultures with *P. salmonis*.

## Discussion

In this study, we demonstrated that *P. salmonis* is able to survive, replicate, and perturb lysosomal activation in infected macrophage-enriched cell cultures. This evasion mechanism associated to phagosomal maturation has been previously reported in bacteria closely related to *P. salmonis*, including *L. pneumophila* and *C. burnetii* (21, 22).

The lysosome is the major degradative compartment of eukaryotic cells. It is a membrane-bound compartment specialized in the degradation and recycling of cellular components. In addition, it has an internal acidic pH that provides an optimal environment for the function of hydrolases, thus facilitating the degradation of macromolecules (41). Multiple endocytic pathways, such as phagocytosis, macropynocitosis, clathrin- and caveolin-dependent and independent endocytosis, import macromolecules from extracellular environment and from the cell's own limiting membrane to be degraded via the lysosomal system. Moreover, the self-catabolic process—known as autophagy—allows the capture and delivery of cytoplasmic macromolecules (i.e., damaged or misfolded proteins) and entire organelles to the lysosome (41).

Macrophages have been described as a phagocyte population that play an important role in specific and non-specific immune responses, owing to their specialized phagocytic capacity, antigen-presenting function, secretion of cytokines, and efficient antimicrobial activity (42, 43). Considering that the macrophages are one of the first cell types which pathogens encounter after their entry into the host, many pathogens are internalized by endocytosis or phagocytosis—intracellular pathways that culminate the degradation in the lysosomal system. In this sense, it is not surprising that some pathogens have developed intracellular immune evasion mechanisms against macrophages (14, 44, 45). In the present study, we observed that *P. salmonis* evades host response mechanisms by reducing the lysosomal acidification and its proteolytic activity in the first hpi. Thus, this pathogen is able to reside in macrophage-enriched cell cultures for 24 hpi and replicate for ≥120 hpi (Figure 1).

Macrophage infection is a common strategy adopted by several intracellular pathogens to colonize and spread into their hosts. This is achieved through interference with normal cell signaling and disruption of the normal responses triggered to eliminate foreign invaders, such as the lysosomal system of degradation. Interference with this system allows pathogen survival and evasion of the host immune response (11, 18, 45). In mammals, a good example is *L. pneumophila*, which presents mechanisms to recruit and manipulate the transport of the phagosome using >300 bacterial genes encoding different proteins (termed effectors) that are secreted from the bacteria to the host cell (46, 47). One of these mechanisms is associated with the prevention of phagosome acidification through the recruitment of Rab1 to the phagosome (48). Acidification of the phagosome is necessary for efficient lysosomal fusion and function. *L. pneumophila* employs several mechanisms to recruit Rab1 to the Legionella-containing vacuole (LCV). Multiple Dot/Icm secreted factors, such as DrrA/SidM, SidD, and LepB function together to manipulate the localization of Rab1 to the LCV, thus cycling the host protein between the active (anchored to the LCV) and inactive states. On the other hand, *L. pneumophila* also manipulates Rab1 independently of recruitment to the LCV through the action of SidC/SdcA, LidA, and AnkX. This redundancy in the mechanisms indicates that Rab1 manipulation by *L. pneumophila* is extremely important for the intracellular survival of this pathogen (49). The molecular mechanisms involved in host response evasion which are modulated by *P. salmonis* remain to be elucidated.

In fish, relatively few pathogens have been reported to resist degradation and replicate within macrophages. Virulent *Edwarsiella tarda* is capable of survival and replication in head kidney phagocytes of blue gourami (*Trichogaster trichopterus*) at least by 6.5 hpi, where induces low levels of reactive oxygen intermediates by infected phagocytes (14, 50). *Photobacterium damselae* subsp. *piscicida* is an intracellular pathogen that can survive and multiply within macrophages of hybrid striped bass, via inhibition of the phagosome-lysosome fusion and replication in vacuoles at least by 18 hpi (*Morone saxatilis* × *white bass M. chrysops*) (14, 51). Similarly, *Mycobacterium* spp. have been found both intact and partially degraded inside phagolysosomes of rainbow trout macrophages, suggesting that some degree of inhibition of the phagosome-lysosome fusion occurs to favor bacterial survival (14, 52). *Renibacterium salmoninarum* survives within macrophages even at 4 dpi, suggesting that this bacterium escapes from the phagosome and resides in a bacteria-containing vesicle with a tightly apposed membrane by interfering with the macrophage-killing pathways (14, 53, 54).

These examples reflect the possibility that in fish, like in mammals, certain pathogens have developed evasion mechanisms against macrophages as part of their life-cycle, using the cell as a shield against cell-mediated and humoral immune responses (44). However, the mechanism used by fish pathogens to resist the macrophage killing-mechanism is currently not understood and requires investigation. In the present study, we detected viable *P. salmonis* in macrophage-enriched cell cultures after 5 dpi and observed a two-fold increase in its detection using qPCR. These results suggest bacterial survival and replication (Figure 2). The increase in bacterial load detected through qPCR at 120 hpi was inconsistent with the FITC-fluorescence intensity observed at the same time points (Figure 1). However, this discrepancy is probably due to a decline in FITC fluorescence, which has been reported to decrease during prolonged incubation times (55). However, this approach allows us to observe *P. salmonis* in the infected cells. These results are consistent with those previously observed in macrophages obtained from rainbow trout head kidneys—in which concurrent destruction and replication were observed at 5 dpi—implying that *P. salmonis* is able to survive and reproduce in functional macrophages (14).

An analysis performed to characterize the function of the lysosome showed that the number of lysosomes in macrophage-enriched cell cultures infected with *P. salmonis* (Figure 3) does not increase in the first 24 hpi. This evidence suggests a delayed response, which is further supported by evidence showing that incubation with inactivated *P. salmonis* resulted in an increase in the number of lysosomes by more than three-fold 6 h post-incubation. An increase in lysosome biogenesis/function has been reported to contribute to limiting intracellular macrophage infection in mice (56). Lysosomes must maintain an acidic luminal pH to activate hydrolytic enzymes and degrade internalized macromolecules (57). Thus, lysosomal acidification is a key environmental mechanism for the correct function of this organelle. Analysis of the lysosomal pH following infection of macrophage-enriched cell cultures with *P. salmonis* showed that at 3 hpi, approximately 70% of lysosomes presented an acidic pH. Interestingly, this percentage diminished at 6 and 24 hpi to values <40%. Conversely, incubation with inactivated *P. salmonis* showed an acidic pH in >75% of lysosomes, reflecting that macrophage-like cells respond to the appropriate stimulus. Previous studies have reported early lysosomal acidification in macrophage-enriched cell cultures infected by *P. salmonis* at 2 hpi, suggesting that the acidification may be the signal that induces the intracellular overexpression of the *P. salmonis dot/icm* genes (11). This *dot/icm* gene expression may be responsible for the observed decrease in the number of lysosomes with an acidic pH, as previously described in *L. pneumophila* (48, 49). The functional consequence of the disruption of lysosomal acidification is a poor hydrolytic activity. In the present study, this was observed by the low levels of degradation of DQ™ Green BSA in macrophage-like cells infected by *P. salmonis* compared with those observed in cells incubated with inactivated *P. salmonis* (Figure 4). However, expression or functional analyses are required to identify which part of the mechanism of assembly or function is targeted by *P. salmonis* during its interference with the lysosomal response.

Collectively, the results showed that infection of Atlantic salmon macrophages with *P. salmonis* induced a limited lysosomal response in the first hpi, which may correspond to a strategy of immune evasion that has not been previously reported. Previous studies have described that *P. salmonis* does not reach lysosomal compartments in which TIVBSS plays a central role (11, 14). Currently, there is limited knowledge available regarding the pathogenetic mechanisms of *P. salmonis*. However, the similarities observed with the phylogenetically closely related bacteria *L. pneumophila* (58) suggest that *P. salmonis* may use a similar mechanism of pathogenesis to those of *L. pneumophila*. Both bacteria target macrophages and disruption of the lysosomal degradation pathways appears to be a hallmark of their infection.

## Data Availability

All datasets generated for this study are included in the manuscript and/or the supplementary files.

## Author Contributions

DP-S and JM-R performed most of the experiments, analyzed the data, prepared the figures, and contributed to the writing of this manuscript. ST, DA, AE, VS-H, BB, and VI performed the experiments. JV, EV-V, AS, and ES reviewed this manuscript. SR-C conceived and supervised the study. SR-C and FR-L wrote this manuscript.

### Conflict of Interest Statement

JM-R, AS, and ES were employed by company Consorcio de Sanidad Acuícola Ictio Biotechnologies S.A. The remaining authors declare that the research was conducted in the absence of any commercial or financial relationships that could be construed as a potential conflict of interest.

## Supplementary Material

Supplementary Figure 1 Viability assay of FITC stained *P. salmonis*. Non-stained *P. salmonis* and FITC stained *P. salmonis* were plated on enriched blood agar. Confirmation that identity of colonies correspond to *P. salmonis* was carried out by specific detection of 16S rDNA by real time PCR. **(A)** FITC-stained *P. salmonis* plated on enriched blood agar. **(B)** Melt curve of the gene amplification obtained by real time PCR from a single colonies of FITC stained *P. salmonis* (red lines) and positive control (gray lines). **(C)** *P. salmonis* plated on enriched blood agar. **(D)** Melt curve of the gene amplification obtained by real time PCR from a single colonies of *P. salmonis* (red lines) and positive control (gray lines).
