# Supplementary figures and images for: Non-lysosomal Activation in Macrophages of Atlantic Salmon (Salmo salar) After Infection With Piscirickettsia salmonis
Source: Front Immunol. 2019 Mar 19;10:434. doi: 10.3389/fimmu.2019.00434 (PMC6433878; doi:10.3389/fimmu.2019.00434)

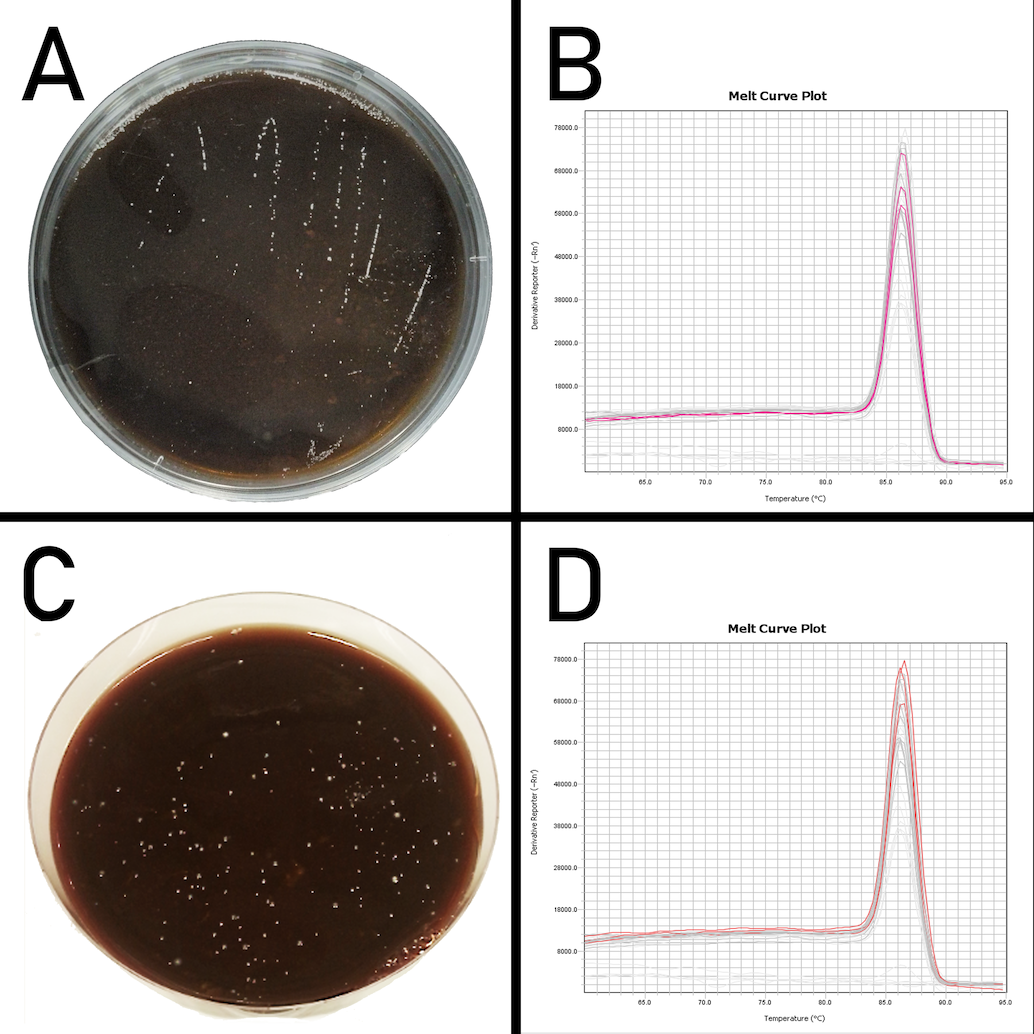

Supplement: Supplementary Figure 1 — Viability assay of FITC stained P. salmonis. Non-stained P. salmonis and FITC stained P. salmonis were plated on enriched blood agar. Confirmation that identity of colonies correspond to P. salmonis was carried out by specific detection of 16S rDNA by real time PCR. (A) FITC-stained P. salmonis plated on enriched blood agar. (B) Melt curve of the gene amplification obtained by real time PCR from a single colonies of FITC stained P. salmonis (red lines) and positive control (gray lines). (C) P. salmonis plated on enriched blood agar. (D) Melt curve of the gene amplification obtained by real time PCR from a single colonies of P. salmonis (red lines) and positive control (gray lines). [file Image_1.TIF]
